# Impact of Fc-gamma receptor IIIA polymorphism on late-onset neutropenia and clinical outcomes in kidney transplant recipients following rituximab induction therapy

**DOI:** 10.1007/s10157-024-02610-7

**Published:** 2025-01-13

**Authors:** Yuki Tashiro, Yoji Hyodo, Satoshi Kitamura, Takuya Fujimoto, Takahito Endo, Shun Nishioka, Naoki Yokoyama, Takuto Hara, Koji Chiba, Hideaki Miyake

**Affiliations:** 1 Division of Urology, Kobe University Graduate School of Medicine, 7-5-2 Kusunoki-cho, Chuo-ku, Kobe, 650-0017 Japan; 2 Department of Urology, Hyogo Prefectural Amagasaki General Medical Center, Amagasaki, Hyogo Japan

**Keywords:** Fc-gamma receptor IIIa, Neutropenia, Polymorphisms, Kidney transplantation, Rituximab

## Abstract

**Background:**

This study aimed to investigate the association between the Fc-gamma receptor IIIA (FCGR3A) 158 polymorphism and clinical outcomes in kidney transplantation (KTx) patients. Specifically, we focused on late-onset neutropenia (LON) in ABO-incompatible (ABOi) or HLA-incompatible (HLAi) KTx recipients who underwent rituximab (RTx) desensitization therapy.

**Methods:**

FCGR3A 158F/V polymorphisms were identified in 85 ABOi or HLAi KTx recipients who underwent RTx desensitization at our institution between April 2008 and October 2021. We analyzed these polymorphism groups in relation to their preoperative background and incidence of LON, infection, and rejection. In addition, we examined the risk factors for LON development.

**Results:**

The following FCGR3A 158F/V polymorphisms were identified: FF genotype (*n* = 45); FV genotype (*n* = 36), and VV genotype (*n* = 4). LON occurred in 25 out of 85 recipients within 1 year after KTx, significantly more frequently in patients with the FCGR3A FV + VV genotype (17/40) than in those with the FF genotype (8/45) (*p* = 0.01). A multivariate analysis identified the V-allele as an independent risk factor for LON (OR, 4.03; 95% CI, 1.38—11.73, *p* = 0.01). However, there were no significant differences in the incidence rates of post-transplant infection and rejection between the FF and FV + VV genotypes.

**Conclusion:**

Recipients with the FCGR3A 158 V-allele were identified as having a higher risk of developing LON following KTx with RTx desensitization therapy. However, the presence of this V-allele did not affect the safety or efficacy of RTx desensitization before KTx.

**Supplementary Information:**

The online version contains supplementary material available at 10.1007/s10157-024-02610-7.

## Introduction

Rituximab (RTx) induction therapy, a CD20 chimeric antibody combined with antibody removal by plasmapheresis, effectively reduces the risk of postoperative rejection in ABO-incompatible (ABOi) and HLA-incompatible (HLAi) kidney transplantation (KTx) recipients, particularly those with high immunological risk [1, 2]. However, despite its benefits, RTx is associated with serious adverse effects, particularly late-onset neutropenia (LON), which can lead to severe infections [3]. Importantly, neutropenia after KTx is associated with an increased risk of graft loss or mortality [4].

A single nucleotide polymorphism (SNP) in the gene encoding Fc-gamma receptor IIIA (FCGR3A; CD16), which is present in natural killer (NK) cells and monocytes/macrophages, represents an allelic variant of amino acid 158 within the IgG-binding domain of the CD16 receptor [5]. The FCGR3A 158F/V polymorphism (rs396991) influences CD16 expression and antibody-dependent cellular cytotoxicity (ADCC) in NK cells [6, 7]. The high-affinity FCGR3A 158 V-allele has been detected in patients with rheumatic diseases and lymphoma who developed LON after treatment with RTx [8–10]. This SNP has also been observed in immunosuppressed KTx recipients. In KTx recipients with chronic active antibody-mediated rejection (caABMR), the FCGR3A VV-genotype was reported to be associated with decreased graft survival and increased glomerulitis scores [11]. In contrast, the presence of the F-allele has been reported to be associated with an increased risk of urinary tract infection (UTI) [12].

However, the association between FCGR3A polymorphism and the occurrence of LON in solid organ transplantation (SOT) recipients after RTx induction therapy remains unclear. This is partly due to RTx as well as other factors such as immunosuppressive agents and cytomegalovirus (CMV) infections, which could cause neutropenia in these patients [13]. In addition, the association between this SNP and the frequency of post-transplant infection and rejection, as well as graft survival in SOT recipients after RTx desensitization, remains unknown. Therefore, we aimed to evaluate the association between FCGR3A 158F/V polymorphism and clinical outcomes, including LON, in KTx recipients after RTx induction therapy.

## Materials and methods

### Study population and design

This study included patients who underwent KTx with induction RTx therapy at Kobe University Hospital, Japan, between April 2008 and October 2021. Among 109 patients who underwent ABOi or HLAi KTx during this period, three patients had already died from causes unrelated to renal function, and 21 patients were excluded due to lack of consent or transferred to other hospitals. Ultimately, 85 patients were included in this study. In this study, HLAi KTx was defined as maximal donor-specific antibody (DSA) mean fluorescence intensity (MFI) ≥ 1000, irrespective of the complement-dependent cytotoxicity crossmatch (CDCXM) and flow-cytometric crossmatch (FCXM) results. This study was approved by the Research Ethics Committee of Kobe University Hospital (No. B220142). Written informed consent for the use of blood samples was obtained from all participants.

### Immunosuppressive therapy

All patients received induction therapy with RTx, calcineurin inhibitors (CNI), mycophenolate mofetil (MMF), methylprednisolone, and basiliximab. Basiliximab was administered on the day of KTx and 4 days after KTx. MMF (20–30 mg/kg) was administered orally from 2 weeks before KTx, and CNI administration began 1 week before KTx, with the dosage adjusted according to individual patient factors and trough levels monitored to optimize immunosuppression. The methylprednisolone regimen involved oral administration starting at 16 mg/day 1 week before KTx, a bolus dose of 500 mg on the day of KTx, and tapering to 4 mg/day by 4 weeks after KTx. If no apparent rejection or recurrence of the underlying disease was observed in the kidney biopsy 3 months after KTx, the patients began everolimus as maintenance therapy and the target trough levels of CNI were decreased. RTx was administered twice, 14 days and 1 day before KTx. The standard dose was 100 mg/m^2^ per session, with a total of 200 mg/m^2^. After 2015, patients with anti-A/B antibody titers of ≥ 64 or HLAi received 150 mg/m^2^ RTx (total RTx dose, 300 mg/m^2^). All patients underwent 1 or 2 sessions of double filtration plasmapheresis (DFPP) and 1 or 2 sessions of plasma exchange (PEX) before the second RTx administration, depending on the anti-ABO blood type antibody titer or DSA positivity. Since 2019, HLAi KTx recipients have received IVIG (1 g/kg) for 4 days before KTx.

### Infections

CMV antibody tests were conducted for all KTx recipients and donors before KTx, with CMV antigenemia assays performed weekly for 3 months post-KTx and monthly for the first year thereafter. CMV viremia was defined as the presence of CMV pp65 antigen (C7-HRP)-positive cells at a level of ≥ 1/50000 leukocytes. CMV treatment was administered preemptively, and the administration of valganciclovir or ganciclovir for CMV antigenemia positivity was determined at the discretion of the attending physician. Fever episodes ≥ 38 °C, suspected CMV gastrointestinal involvement, and endoscopic findings were classified as CMV disease. No prophylactic CMV treatment was administered. The incidence of UTIs and bloodstream infections (BSIs) within 12 months post-KTx was also recorded.

### Neutropenia and granulocyte colony stimulating factor (G-CSF) treatment

In this study, neutropenia was defined as an absolute neutrophil count (ANC) < 1500/μL (grade ≥ 2 based on the National Cancer Institute’s Common Terminology Criteria for Adverse Events [CTCAE v5.0]) occurring within 1 year after KTx. LON was defined as neutropenia occurring more than 4 weeks after the last administration of RTx, excluding patients with CMV infection (i.e., CMV viremia or those on anti-CMV drugs). Neutropenia occurring > 1 month after the resolution of CMV viremia and discontinuation of anti-CMV drugs, and without other causes was included as LON. Patients with severe LON (ANC < 1000/μL) received G-CSF until improvement, whereas those who developed severe neutropenia during CMV infection had their dose of MMF or anti-CMV drugs reduced or discontinued.

### Post-transplant kidney biopsy

Protocol biopsies were performed at 3 months and at 1, 3, 5, 7, and 10 years post-KTx for all patients, with episode biopsies conducted when rejection was suspected. Acute rejection was determined according to Banff criteria for each era.

### FCGR3A genotyping

Genomic DNA was extracted from whole blood samples using a NucleoSpin Blood Kit (Macherey–Nagel GmbH & Co. KG, Düren, Germany). The FCGR3A 158F/V polymorphism (rs396991) was genotyped based on allelic discrimination using Taqman SNP Genotyping assay (C_25815666_10; Thermo Fisher Scientific, Waltham, Massachusetts, USA) and Taqman Universal PCR Master Mix. A 7500 Real-Time PCR System (Applied Biosystems, Waltham, Massachusetts, USA) was used to perform polymerase chain reaction to determine the genotypes. All samples were run in duplicate.

### Statistical analysis

Statistical analyses were performed using JMP Pro (ver. 17 for Mac; SAS Institute). Categorical variables were compared using the Chi-square test or Fisher’s exact test. Continuous variables were compared using Student’s *t* test or the Mann–Whitney *U* test. A logistic regression model was used to test the influence of continuous or categorical variables on the categorical outcomes. Multivariate logistic regression analysis was conducted to identify variables independently associated with neutropenia. Variable selection was conducted based on the Akaike Information Criterion (AIC) and established evidence from the literature to improve model fit [14–17]. We assessed multicollinearity among the independent variables by calculating the variance inflation factors (VIF). VIF > 3 was considered indicative of potential multicollinearity and was, therefore, excluded. The Kaplan–Meier method with log-rank statistics was used to analyze differences in graft survival after KTx between the FCGR3A FF-genotype and the FV + VV-genotype. Statistical significance was set at *p* < 0.05.

## Results

### Baseline characteristics

Eighty-five patients were included in the study. SNP analysis revealed FCGR3A 158 genotypes of FF, FV, and VV in 45 (52.9%), 36 (42.4%), and four (4.7%) recipients, respectively. The frequency of the V-allele was 25.9%, which is consistent with the Hardy–Weinberg equilibrium, indicating that allele frequencies are stable within this population [18]. Table 1 presents the baseline characteristics of the patients with the FCGR3A 158FF-genotype and FV + VV-genotypes. Among these patients, 69 (81.2%) underwent ABOi KTx, and 24 (28.2%) underwent HLAi KTx. The median follow-up duration was 81.3 (58.2–121.6) months for the FCGR3A FF-genotype group and 66.4 (52.3–110.0) months for the FV + VV-genotype group, respectively (*p* = 0.34). Although no statistically significant differences were observed in the baseline characteristics of the groups (Table 1), the FF-genotype group tended to have a higher incidence of HLAi KTx, especially a higher proportion of FCXM-positive cases, and CMV high-risk status, defined as CMV-positive donors and CMV-negative recipients, who are at increased risk for CMV infection after KTx.

**Table 1:** Characteristics of recipients with FCGR3A 158 FF-genotype and FV + VV-genotype

	FF (*n* = 45)	FV + VV (*n* = 40)		*p* value
Follow-up duration, months, median (IQR)	81.3 (58.2 – 121.6)	66.4	(52.3 – 110.0)	0.34
Recipient age, years, median (IQR)	48.0 (36.0 – 61.0)	42.0	(30.0 – 54.0)	0.22
Donor age, years, median (IQR)	56.5 (47.5 – 65.0)	58.5	(49.3 – 67.0)	0.38
Recipient male sex, n (%)	28 (62.2)	24	(60.0)	0.83
BMI before KTx, kg/m^2^, median (IQR)	21.3 (18.5 – 23.7)	21.8	(17.9 – 24.1)	0.96
Deceased donor, n (%)	1 (2.2)	1	(2.5)	1
Preemptive KTx, n (%)	21 (46.7)	18	(45.0)	0.88
Dialysis period, months, median (IQR)	5.1 (0 – 42.1)	7.4	(0 – 31.5)	0.77
ABO-incompatible KTx, n (%)	36 (80.0)	33	(82.5)	0.79
Re-transplantation, n (%)	2 (4.4)	2	(5.0)	1
HLA-incompatible KTx, n (%)	16 (35.6)	8	(20.0)	0.15
CDCXM positive, n (%)	2 (4.4)	0	(0)	0.50
FCXM positive, n (%)	16 (35.6)	6	(15.0)	0.05
HLA-DSA only positive, n (%)	0 (0)	2	(5.0)	0.22
HLA mismatch in A, B, DR, median (IQR)	4.0 (3.0– 5.5)	3.0	(3.0 – 5.0)	0.25
Origin of ESRD, n (%)				0.02
CGN	16 (35.6)	14	(35.0)	
IgAN	8 (17.8)	5	(12.5)	
DMN	7 (15.6)	2	(5.0)	
ADPKD	4 (8.9)	1	(2.5)	
FSGS	0 (0.0)	7	(17.5)	
Renal sclerosis	1 (2.2)	3	(7.5)	
Unknown	0 (0.0)	2	(5.0)	
Others	9 (20.0)	6	(15.0)	
Total RTx dose before KTx, n (%)				0.75
100 mg/m^2^	1 (2.2)	1	(2.5)	
200 mg/m^2^	25 (55.6)	25	(62.5)	
300 mg/m^2^	19 (42.2)	14	(35.0)	
IVIG before KTx, n (%)	3 (6.7)	0	(0.0)	0.24
ANC before RTx therapy (*10^3^/μL), median (IQR)	3.6 (3.0 – 4.3)	3.6	(2.7 – 4.5)	0.73
CD19^+^ cells on the day before KTx (%), median (IQR)	0.1 (0 – 0.2)	0.1	(0 – 0.2)	0.99
CMV D + R-, n (%)	10 (22.7)	4	(10.3)	0.15
Tacrolimus maintenance, n (%)	45 (100)	38	(95.0)	0.22
Everolimus maintenance, n (%)	25 (55.6)	29	(72.5)	0.12
Trough level of tacrolimus (ng/mL), median (IQR)				
6 months after KTx	5.2 (4.5 – 5.8)	5.1	(4.2 – 6.0)	0.97
12 months after KTx	4.6 (3.9 – 5.2)	4.0	(3.3 – 5.0)	0.07
Trough level of everolimus (ng/mL), median (IQR)				
6 months after KTx	5.0 (3.8 – 5.9)	5.2	(4.2 – 5.8)	0.96
12 months after KTx	5.2 (4.5 – 5.7)	4.9	(3.5 – 5.9)	0.52
Dose of MMF (mg/kg), median (IQR)				
Initial dose	21.7 (19.2 – 24.1)	21.3	(18.8 – 24.1)	0.74
6 months after KTx	17.9 (14.2 – 22.2)	19.2	(14.3 – 22.6)	0.61
12 months after KTx	17.5 (13.6 – 21.7)	19.2	(14.4 – 22.6)	0.42
eGFR (mL/min/1.73m^2^), median (IQR)				
6 months after KTx	48.8 (44.4 – 55.5)	44.5	(37.0 – 57.4)	0.31
12 months after KTx	48.0 (41.6 – 52.5)	45.9	(38.2 – 64.3)	0.87

Data are presented as medians (IQR) for continuous variables and numbers (%) for categorical variables

BMI, body mass index; CDCXM, complement-dependent cytotoxicity crossmatch; FCXM, flow-cytometric crossmatch; HLA-DSA, donor-specific human leukocyte antibody; ESRD, end-stage renal disease; CGN, chronic glomerulonephritis; IgAN, IgA nephropathy; DMN, diabetic nephropathy; ADPKD, autosomal dominant polycystic kidney disease; FSGS, focal segmental glomerulosclerosis; IVIG, intravenous Immunoglobulin; ANC, absolute neutrophil count; CD19^+^, CD19-positive; CMV D + R-, preoperative cytomegalovirus antibody-seropositive donor and seronegative recipient; eGFR, estimated glomerular filtration rate

### Characteristics of neutropenia and risk factors for neutropenia

LON was observed in 8 cases (17.8%) with the FF-genotype and in 17 cases (42.5%) with the FV + VV-genotype (Table 2). The incidence of LON was significantly higher in the FV + VV-genotype group than in the FF-genotype group (*p* < 0.01). Severe LON (ANC < 1000/μL) was more common in the FV + VV-genotype group, but the difference was not statistically significant (*p* = 0.16). The median time between the final administration of RTx and the onset of LON was 166.0 (106.5–250.0) days. Neutropenia during CMV infection occurred in 20 recipients (44.4%) with the FF-genotype and in 8 recipients (20.0%) with the FV + VV-genotype, with a significant difference between the 2 groups (*p* = 0.017). Among the cases of neutropenia during CMV infection, 17/20 (85%) in the FF-genotype group and 8/8 (100%) in the FV + VV-genotype group were administered anti-CMV drugs, with no significant difference observed between the 2 groups (*p* = 0.07). The dose of MMF at the onset of neutropenia during CMV infection was higher in the FF-genotype group; however, similar to the onset of LON, there was no significant difference between the two groups (Supplementary Table 1). Post-transplant neutropenia was observed in 53 cases (62.3%), with 27 (60.0%) in the FF-genotype group and 26 (65.0%) in the FV + VV-genotype group (*p* = 0.66). Among these, 2 cases with the FF-genotype and 3 cases with the FV + VV-genotype developed both LON and neutropenia during CMV infection. There was also no significant difference in the prevalence of severe neutropenia, defined as an ANC of less than 1000/μL (33.3% vs. 32.5%, *p* = 0.94). Independent risk factors for LON and neutropenia during CMV infection were evaluated (Table 3). The multivariate analysis revealed the presence of the FCGR3A 158 V-allele as an independent risk factor for LON (OR 4.03, 95% CI 1.38 – 11.73, *p* = 0.01). In the univariate analysis, the FCGR3A 158FF-genotype and CMV high-risk status were found to be significantly associated with neutropenia during CMV infection. While these associations were not statistically significant in the multivariate analysis, although they approached statistical significance (*p* > 0.05).

**Table 2:** Impact of FCGR3A polymorphism on post-transplant neutropenia

Neutropenia	FF (*n* = 45)	FV + VV (*n* = 40)	*p* value
LON, n (%)	8 (17.8)	17 (42.5)	0.013^a^
LON (ANC < 1000/μL), n (%)	5 (11.1)	9 (22.5)	0.16
During CMV infection status, n (%)	20 (44.4)	8 (20.0)	0.017^a^

The table shows the number of neutropenia and LON in the FCGR3A 158 FF-genotype and FV + VV-genotype

ANC, absolute neutrophil count

^a^ Statistically significant

**Table 3:** Association of baseline kidney transplant recipients’ characteristics with (A) LON and (B) neutropenia during CMV infection in univariate and multivariate analysis

(A)	Univariate analysis			Multivariate analysis			VIF
	OR	95% CI	*p* value	OR	95%CI	*p* value	
Recipient age (for 1 year increase)	1.02	0.99–1.05	0.14	1.03	0.99–1.07	0.13	1.07
Dialysis period (for 1 month increase)	1.00	0.99–1.01	0.97				
ABO-incompatible KTx	1.31	0.38–4.55	0.77				
HLA-incompatible KTx	1.69	0.62–4.61	0.30				
Total RTx dose > 200 mg/m^2^	1.71	0.66–4.41	0.26				
IVIG before KTx	1.21	0.10–13.96	1.00				
CMV D + R-	0.36	0.07–1.72	0.33				
Initial dose of MMF > 25 mg/kg	2.72	0.84–8.85	0.11	2.77	0.80–9.64	0.11	1.02
Tacrolimus maintenance (vs CyA)	0.41	0.02–6.77	0.50				
Everolimus maintenance (vs Non)	0.64	0.24–1.65	0.35				
With FCGR3A 158 V-allele	3.42	1.27–9.19	0.01^a^	4.03	1.38–11.73	0.01^a^	1.06

(B)	Univariate analysis			Multivariate analysis			VIF
	OR	95% CI	*p* value	OR	95% CI	*p* value	
Recipient age (for 1 year increase)	1.02	0.99–1.05	0.24				
Dialysis period (for 1 month increase)	1.00	0.99–1.01	0.71				
ABO-incompatible KTx	1.10	0.34–3.54	0.87				
HLA-incompatible KTx	1.70	0.64–4.55	0.28	2.22	0.74–6.72	0.16	1.09
Total RTx dose > 200 mg/m^2^	1.29	0.51–3.23	0.59				
IVIG before KTx	1.02	0.09–11.73	1.00				
CMV D + R-	3.27	1.00–10.52	0.04^a^	3.54	0.98–12.73	0.05	1.04
Initial dose of MMF > 25 mg/kg	1.14	0.34–3.80	1.00				
Tacrolimus maintenance (vs CyA)	-	-	-				
Everolimus maintenance (vs Non)	1.05	0.41–2.69	0.92				
With FCGR3A 158FF-genotype	3.20	1.21–8.46	0.02^a^	2.55	0.92–7.08	0.07	1.06

VIF, variance inflation factor; OR, odds ratio; CI, confidence interval; IVIG, intravenous Immunoglobulin; CMV D + R-, preoperative cytomegalovirus antibody-seropositive donor and seronegative recipient; CyA, cyclosporine

ORs and 95% CIs were calculated using the Chi-square test or Fisher’s exact test for categorical variables and univariate logistic regression analysis for continuous variables. Subsequently, a multivariate logistic regression analysis was conducted for the three explanatory variables

^a^ Statistically significant

### Post-transplant infectious episodes

Table 4 shows the prevalence of post-transplant CMV infections in the FF-genotype and FV + VV-genotype groups. The incidence rates of CMV viremia and disease after KTx did not differ significantly between the two groups. The median time between KTx and the first CMV infection was 39.0 (18.0–55.0) days in the FF-genotype group and 41.0 (29.0–63.0) days in the FV + VV-genotype group, with no significant difference. In addition, early UTIs and BSIs within 1 year of KTx were compared between the 2 groups (Table 4). Recipients with the FF-genotype had a slightly higher incidence of UTIs within 1 year after KTx, but the difference was not statistically significant. Importantly, none of the recipients in either group developed febrile neutropenia, which is difficult to treat.

**Table 4:** The incidence of infections by FCGR3A polymorphism

	FF (n = 45)	FV + VV (n = 40)	OR	95% CI	*p* value
CMV viremia, n (%)	33 (73.3)	24 (60.0)	0.55	0.22–1.36	0.19
CMV disease, n (%)	5 (11.1)	3 (7.5)	0.64	0.14–2.91	0.72
Anti-CMV drug administration, n (%)	24 (53.3)	12 (30.0)	0.38	0.15–0.92	0.03^a^
Neutropenia during anti-CMV drug administration, n (%)	17 (37.8)	8 (20.0)	0.41	0.15–1.10	0.07
UTIs within 12 months, n (%)	17 (37.8)	8 (20.0)	0.41	0.15–1.10	0.10
BSIs within 12 months, n (%)	3 (6.7)	3 (7.5)	1.14	0.22–5.97	1

Fisher’s exact test was used to estimate odds ratios (OR) and 95% confidence intervals (CI)

### Allograft rejection and graft survival

**Fig. 1:**
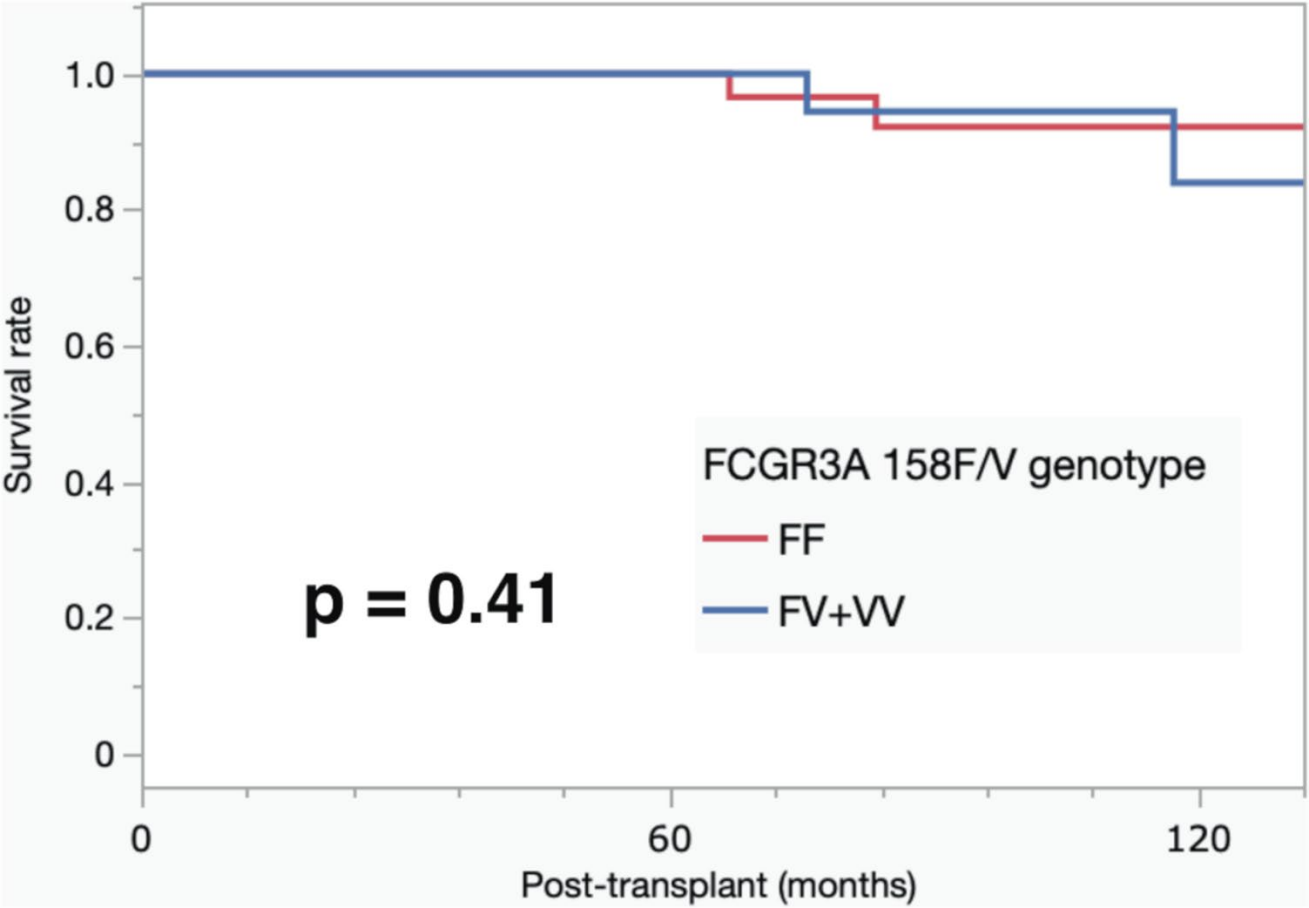
The 10-year graft survival. The survival rate was determined using the Kaplan–Meier method. The 10-year graft survival rates were 92.6% and 84.0% in recipients with the FCGR3A 158FF-genotype and FV + VV-genotype, respectively (*p* = 0.41)

The prevalence of acute antibody-mediated rejection (acute ABMR) and T-cell-mediated rejection (TCMR) was similar between the 2 groups. Acute ABMR occurred in 13.3% (6/45) of the patients in the FCGR3A FF-genotype group and in 15.0% (6/40) of the patients in the FV + VV-genotype group. Similarly, TCMR was observed in 4.4% (2/45) of patients in the FF-genotype group and in 2.5% (1/40) of patients in the FV + VV-genotype group (Table 5). During the follow-up period, two patients died due to graft function, but there was no significant difference in patient survival between the two groups. Furthermore, no significant difference in graft survival rate was observed between the two groups (*p* = 0.41) (Fig. 1).

**Table 5:** The incidence of ABMRs and TCMRs within 3 months after kidney transplantations by FCGR3A polymorphism

	FF (*n* = 45)	FV + VV (*n* = 40)	OR	95% CI	*p* *p* value
acute ABMR, n (%)	6 (13.3)	6 (15.0)	1.15	0.34–3.89	1
TCMR, n (%)	2 (4.4)	1 (2.5)	0.55	0.05–6.32	1

Fisher’s exact test was used to estimate odds ratios (OR) and 95% confidence intervals (CI). *TCMR* T-cell-mediated rejection

## Discussion

In this study, we investigated the influence of FCGR3A variants on the clinical outcomes of KTx recipients receiving RTx as a desensitization therapy. The present study demonstrated three main findings: 1) the FCGR3A 158 V-allele was identified as an independent risk factor for LON in KTx recipients treated with RTx. 2) Univariate analysis showed a significant association between the FCGR3A 158FF-genotype and increased neutropenia rates during CMV infection. 3) No significant differences were observed in the rates of post-transplant UTI or rejection between the 158FF and FV + VV-genotype groups.

The present study revealed that the FCGR3A 158 V-allele was an independent risk factor for LON in KTx recipients desensitized with RTx, a chimeric monoclonal antibody that depletes CD20-positive B cells through different mechanisms, primarily ADCC by NK cells [19, 20]. The FCGR3A 158F/V polymorphism alters CD16 expression and IgG affinity. This change impacts the ADCC function^5^. Previous studies on lymphomas and rheumatic diseases have linked the 158VV-genotype or V-allele to LON [8–10, 21, 22]. One hypothesis is that the higher IgG affinity of the V-allele results in deeper B-cell depletion through ADCC, which may also impact granulocyte hematopoiesis [10]. The incidence of post-transplant neutropenia after ABOi or HLAi KTx desensitized with RTx has been reported to be 42–48% [15, 23]. However, no significant relationship between this SNP and LON has been reported in the context of SOT. This is because multiple factors, such as plasmapheresis, immunosuppressive agents, such as tacrolimus and MMF in maintenance regimens, and CMV infections are thought to contribute to post-transplant neutropenia [15, 23–25]. However, the present study demonstrated that the FCGR3A 158 V-allele is independently associated with the development of LON in KTx recipients with RTx desensitization.

To focus on the relationship between the FCGR3A polymorphism and RTx-induced LON, we excluded cases of neutropenia during CMV infection from the definition of LON in this study. However, a univariate analysis showed a significant association between the FCGR3A 158FF-genotype and an increased frequency of neutropenia during CMV infection. However, this association was not observed in the multivariate analysis adjusted for the preoperative risk of CMV infection and HLAi KTx. Although the differences were not statistically significant, the FF-genotype group tended to have more cases of high-risk CMV infection or more HLAi KTx than the FV + VV-genotype group. CMV infection can directly cause neutropenia, and ganciclovir and valganciclovir have been reported as significant causes of drug-induced neutropenia [13, 26–29]. Notably, 25 cases of neutropenia due to CMV infection occurred during the administration of anti-CMV drugs. In this study, the initial MMF dose and trough levels of tacrolimus 6 and 12 months after KTx were significantly higher in the HLAi KTx group than in the HLA-compatible group (Supplementary Table 2). The FF-genotype group also had a higher proportion of HLAi KTx, which may have led to arbitrary increases in immunosuppressive agents relative to the FV + VV-genotype group. These factors explain the significant association between the FF-genotype group and neutropenia during CMV infection observed in the univariate analysis.

In this study, no significant difference was observed between the two groups in terms of post-transplant UTIs or rejection. Previous studies have reported contrasting results. One study focused on liver transplant recipients and compared those with the FCGR3A VV-genotype to those with the FCGR3A F-allele [30]. A significantly lower incidence of post-transplant BSIs was found in the former group. Another study from the same group reported a higher incidence of UTIs among KTx recipients with the FCGR3A F-allele [12]. In addition, the presence of the FCGR3A V-allele has been reported to significantly increase the frequency of peritubular capillaritis, a characteristic of ABMR, due to the increased recruitment of immune cells in the peritubular capillaries [31]. The FCGR3A VV-genotype has been shown to be associated with decreased 3-year graft survival and more severe glomerulitis in recipients with chronic active ABMR [11]. A possible reason for the discrepancy between these previous findings and our study results may be the relatively small cohort size. In addition, multiple factors, such as differences in immunosuppression and surgical techniques, may contribute to variations in the infection or rejection rates. Furthermore, the inclusion of patients who received RTx in our cohort, as opposed to the absence of KTx desensitized with RTx in the previous two cohorts, may explain the potential suppression of ABMR development and decreased graft survival in our study.

This study has 2 main limitations: 1) we did not include KTx recipients who did not receive RTx induction therapy, so it remains unclear whether FCGR3A polymorphisms influence neutropenia risk during CMV infection without RTx. 2) The small number of recipients with the FCGR3A 158VV-genotype limits the generalizability of our findings. Owing to geographic variations in allele frequencies, a larger cohort is needed to draw more definitive conclusions.

In conclusion, our findings suggest that the FCGR3A 158 polymorphism is significantly associated with LON development following KTx and RTx desensitization therapy. The V-allele was associated with a higher risk of LON, but not with an increased risk of UTIs or rejection episodes, suggesting a relatively favorable clinical outcome in these aspects.

## Supplementary Information

Below is the link to the electronic supplementary material.

Supplementary file1 (DOCX 21 KB)

## Data Availability

The datasets generated and/or analyzed in the current study are available from the corresponding author upon reasonable request.

## Abbreviations

ABOi: Blood group ABO-incompatible
ADCC: Antibody-dependent cellular cytotoxicity
BSI: Bloodstream infection
ABMR: Antibody-mediated rejection
CMV: Cytomegalovirus
CNI: Calcineurin inhibitor
HLAi: Human leukocyte antigen-incompatible
FCGR3A: Fc-gamma receptor IIIA
KTx: Kidney transplantation
LON: Late-onset neutropenia
MMF: Mycophenolate mofetil
RTx: Rituximab
SNP: Single nucleotide polymorphism
UTI: Urinary tract infection
