# Supplementary material for: Impact of Fc-gamma receptor IIIA polymorphism on late-onset neutropenia and clinical outcomes in kidney transplant recipients following rituximab induction therapy
Source: Clin Exp Nephrol. 2025 Jan 13;29(5):681–9. doi: 10.1007/s10157-024-02610-7 (PMC12049374; doi:10.1007/s10157-024-02610-7)
Supplement: Supplementary file 1 — Supplementary file1 (DOCX 21 KB) [file 10157_2024_2610_MOESM1_ESM.docx]

Supplementary Table 1. Comparison of dose of MMF at the onset of LON or neutropenia during CMV infection between the FCGR3A 158FF-genotype and FV+VV-genotype.

|  | FF (n=45) | | FV+VV (n=40) | | p-value |
| --- | --- | --- | --- | --- | --- |
| Dose of MMF (mg/kg), median (IQR) |  |  |  |  |  |
| At the onset of LON | 21.7 | (14.6 – 25.5) | 20.1 | (17.5 – 24.7) | 0.87 |
| At the onset of neutropenia during CMV infection | 19.2 | (16.6 – 23.6) | 11.0 | (9.4 – 27.3) | 0.08 |

Data are presented as the median (IQR).

^a^ Statistically significant.

Supplementary Table 2. Comparison of trough levels of tacrolimus, everolimus, and dose of MMF between HLA-incompatible and compatible cases.

|  | HLA-incompatible  (n = 24) | | HLA-compatible  (n = 61) | | p-value |
| --- | --- | --- | --- | --- | --- |
| Trough level of Tacrolimus (ng/mL), median (IQR) |  |  |  |  |  |
| 6 months after KTx | 5.5 | (4.8 – 6.5) | 5.1 | (4.1 – 5.8) | 0.02^a^ |
| 12 months after KTx | 5.0 | (4.4 – 6.2) | 4.0 | (3.5 – 4.8) | <0.01^a^ |
| Trough level of Everolimus (ng/mL), median (IQR) |  |  |  |  |  |
| 6 months after KTx | 5.0 | (4.3 – 5.9) | 5.0 | (4.1 – 5.7) | 0.72 |
| 12 months after KTx | 3.9 | (3.9 – 5.7) | 5.2 | (4.3 – 5.9) | 0.34 |
| Dose of MMF (mg/kg), median (IQR) |  |  |  |  |  |
| Initial dose | 23.5 | (19.6 – 28.3) | 21.1 | (18.6 – 22.7) | 0.046^a^ |
| 6 months after KTx | 20.0 | (15.5 – 25.0) | 18.9 | (13.6 – 22.2) | 0.22 |
| 12 months after KTx | 20.0 | (15.5 – 25.0) | 17.7 | (13.6 – 21.7) | 0.12 |

Data are presented as the median (IQR).

^a^ Statistically significant.
